# Construction of a Prognostic Model for Predicting Colorectal Cancer Prognosis and Response to Immunotherapy Based on Cuproptosis-Associated lncRNAs

**DOI:** 10.1155/2023/2733232

**Published:** 2023-03-15

**Authors:** Yi Yang, Xiaoli Wang, Jin Lu, Zhiyong Dong, Ruixiang Hu, Wenhui Chen, Songhao Hu, Guanhua Lu, Biao Huang, Shiliang Dong, Lu Wang, Cunchuan Wang

**Affiliations:** ^1^Department of Bariatric Surgery, The First Affiliated Hospital of Jinan University, Guangzhou, Guangdong, China; ^2^Jinan University Institute of Obesity and Metabolic Disorders, Guangzhou, Guangdong, China; ^3^West General Department, The First Affiliated Hospital of Jinan University, Guangzhou, China; ^4^Key Laboratory of Computational Medicine and Intelligent Health of Anhui Higher Education Institutes, Bengbu Medical College, Bengbu, Anhui, China; ^5^Institute of Precision Cancer Medicine and Pathology, Jinan University Medical College, Guangzhou, Guangdong, China

## Abstract

Colorectal cancer (CRC) is a common and highly lethal gastrointestinal malignancy. Immunotherapy has shown positive efficacy in the treatment of CRC; however, only a minority of patients benefit from immunotherapy. The aim of this study is to construct a cuproptosis-related lncRNA (CRLs) risk score model to predict the prognosis and immune infiltration of CRC patients. Firstly, we synthetically analyzed 19 cuproptosis-related genes (CRGs) from CRC samples derived from the TCGA and obtained 33 CRLs that were significantly associated with prognosis. Next, we defined three cuproptosis modification patterns via consensus clustering analysis (C1, C2, and C3). Further analysis showed that there were significant differences in the abundance of B cells, NK cells, fibroblasts, monocytes, CD8^+^ cells, bone marrow dendritic cells, and cytotoxic lymphocytes in different clusters. In addition, the LASSO regression screened out 6 individual CRLs (AC009315.1, PLS3-AS1, ZEB1-AS1, AC007608.3, AC010789.2, and AC010207.1) closely related to the prognosis of CRC. We found that the low-risk group had better survival prognoses in patients. Furthermore, the high-risk group had lower immune scores and exhibited lower CD8^+^ T cell infiltration. Moreover, the low-risk group had lower immune exclusion, immune dysfunction and TIDE scores than the high-risk group. Interestingly, the lncRNAs in our risk model were positively associated with most immune checkpoints. CD274 (PD-L1), CTLA4, and HAVCR2 (TIM3) were positively correlated with risk scores. Moreover, MSI-H patients had lower risk scores than MSI-L patients, and IPS scores were significantly higher in the low CRLs score group. In conclusion, we constructed a novel risk score model with6 lncRNAs related to cuproptosis, which may be a potential biomarker for evaluating the prognosis and immune treatment for CRC.

## 1. Introduction

In the past, surgery and chemotherapy were the main treatment methods for colorectal cancer (CRC) [1]. In recent years, with the advent of immunotherapy, targeted therapy and other treatment strategies, the prognosis of colorectal cancer patients has been significantly improved [2]. However, the prognosis of patients with advanced CRC remains poor, largely due to the lack of highly specific prognostic biomarkers [3]. So far, the TNM staging system is the most commonly used prognostic indicator in clinical practice, but its overall specificity is insufficient [4]. Therefore, it is crucial to explore more sensitive and specific markers for the prognosis of CRC.

Cuproptosis is a newly discovered form of programmed cell death, which is different from known programmed cell death such as apoptosis, ferroptosis, pyroptosis, and necroptosis; relies on intracellular overload of copper ions to cause cellular death. Excessive respiration produces cytotoxicity and eventually induces cell death [5]. Recent studies have shown that cuproptosis regulation is involved in the development and response to therapy of multiple tumor types [6–8]. Numerous proteins, such as CDKN2A, FDX1, DLD, DLAT, LIAS, GLS, LIPT1, MTF1, PDHA1, and PDHB, have been identified that affect tumor cell proliferation and migration and are associated with cuproptosis [9]. Therefore, revealing the occurrence and development mechanism of cuproptosis may provide positive help for the treatment of CRC.

In recent years, immunotherapy has emerged as a promising alternative therapy for CRC patients. However, due to tumor heterogeneity, only a minority of patients benefit from immunotherapy [10]. Given the evidence suggests that intratumoral infiltrating leukocytes are closely associated with the efficiency of immune responses, including in CRC [11]. Therefore, the discovery and identification of novel immune-related gene targets are crucial to accurately predict the immune response of CRC.

LncRNAs are RNAs containing more than 200 nucleotides that cannot be translated into proteins [12]. LncRNAs play an important role in the occurrence and progression of various solid cancers, including CRC [13–15]. More interestingly, a series of prognostic models constructed based on public databases and by analyzing the expression of lncRNAs showed excellent predictive ability [16, 17]. A prognostic model based on a collection of various regulatory functions in tumors may be a positive direction for the exploration of prognostic markers in the future. However, there has been no report on the construction of a prognostic model based on cuproptosis-related lncRNAs (CRLs).

In this study, we obtained RNA-sequencing (RNA-seq) data from the TCGA database and identified 6CRLs significantly associated with prognostic and then developed a prognostic model. In addition, we verified the CRLs model with training and validation cohort and explored its underlying mechanisms through enrichment analysis. Finally, we assessed the relationship between risk scores and immune cell infiltration, drug sensitivity, and immunotherapy efficacy. Our findings will help predict the prognosis of colorectal cancer patients and provide references for clinical immunotherapy.

## 2. Materials and Method

### 2.1. Data Collection and Correlation Analysis

The Cancer Genome Atlas (TCGA) database was used to retrieve the RNA transcriptome dataset and the associated CRC clinical data. Genes were divided into protein-coding genes and lncRNA genes based on information from the annotated human genome. Additionally, the levels of 19 cuproptosis-related genes (CRGs) expression were evaluated. To evaluate the relationship between lncRNAs and CRGs, we employed Pearson correlation coefficients. CRLs were those with an absolute correlation coefficient of >0.4 and a *p* value less than 0.001. After that, patients were split into the training group and the validation group. The data that were retrieved were then used for bioinformatics analysis.

### 2.2. Construction of Risk Model

To find lncRNA predictive characteristics connected to cuproptosis in the training data set, univariate Cox regression analysis and minimal absolute shrinkage and selection operator (lasso) penalized Cox regression analysis were utilized. Each CRC patient's risk score was determined using the following formula: Risk score is equal to Expi *∗* i, where Expi and bi are the expression and coefficient of each lncRNA, respectively.

### 2.3. Estimation of Tumor-Microenvironment Cell Infiltration

In this study, we applied the method of Cell type Identification By Estimating Relative Subsets Of RNA Transcripts (CIBERSORT) to quantify 22 types of immune cells in the tumor and normal tissue [18]. We also applied the microenvironmental cell population counter (MCPcecther) method using the R package “MCPcether” to quantify the absolute abundance of eight immune cell populations and two stromal cell populations in tumor tissues from RNA-seq data.

### 2.4. Prediction of Small Molecule Drugs

The “limma” R package was used to find differentially expressed genes (DEGs) between high- and low-risk groups. Then, in order to identify which potential target chemicals would be helpful, we submitted the first 1000 DEGs to the CMAP database [19].

### 2.5. Statistical Analysis

R software (version 4.1.3, available at https://www.r-project.org) was used for computational and statistical analyses. Their response to immunotherapy was compared using the Wilcoxon rank sum test. The distinctions between the high- and low-risk categories were ascertained using Kaplan-Meier curves and log-rank testing. *p* values under 0.05 were regarded as statistically significant for all analyses.

## 3. Results

### 3.1. Identification of Cuproptosis-Related lncRNAs in CRC Patients

**Figure 1:**
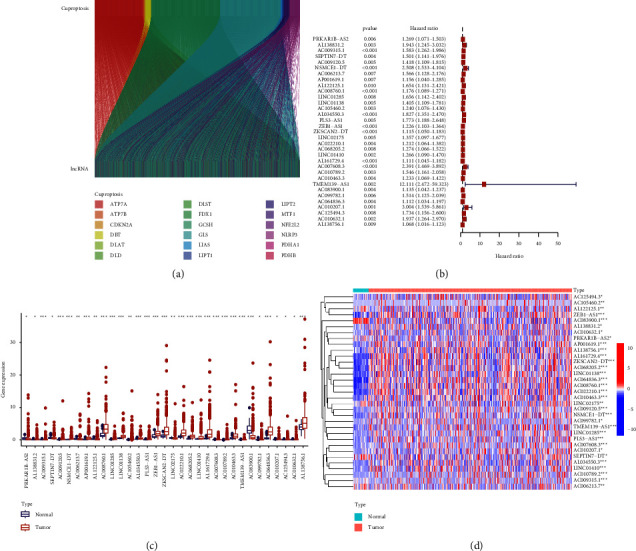
Selection of cuproptosis-related lncRNA in CRC. (a) The Sankey diagram shows the associations between cuproptosis-related lncRNAs and mRNAs. (b) The Forest plot shows 33 lncRNAs with hazard ratios (95% confidence intervals) and *p*-values for their association with CRC prognosis based on univariate Cox proportional-hazards analysis. (c) Histogram of expression levels of 33 lncRNAs in CRC tissues and paired normal tissues. (d) Heatmap of expression levels of 33 lncRNAs in CRC tissues and paired normal tissues.

We first analyzed the CRC mRNA dataset from the TCGA database and obtained the expression profiles of 19 CRGs and 16,876 lncRNAs. Next, we screened out 2450 CRLs by Pearson correlation analysis (|*R*| > 0.4, *p* < 0.001) (Figure 1(a)). We further performed coexpression and univariate Cox regression analysis and obtained 33 CRLs that were significantly associated with prognosis (Figure 1(b)). In addition, we compared the expression of the obtained 33 CRLS in tumor tissue and normal tissue, and the results showed that there were significant differences in their expression levels (Figures 1(c) and 1(d)).

### 3.2. Cuproptosis-Related Genotyping and GSVA Analysis

**Figure 2:**
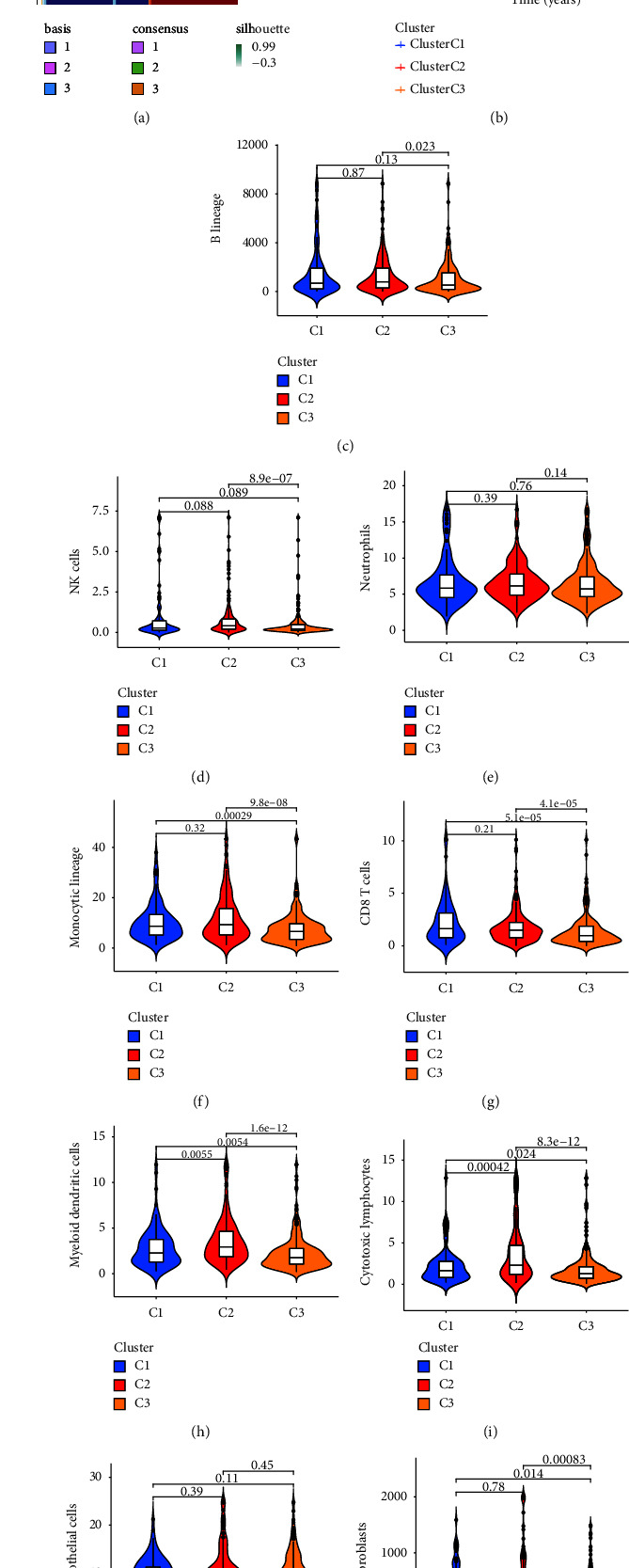
Consensus clustering and the different immune profiles between tree clusters. (a) Consensus map of NMF clustering. (b) Overall survival curves of the three molecular subtypes. (c–k) Comparisons of the abundance of infiltrating immune function between C1, C2 and C3.

**Figure 3:**
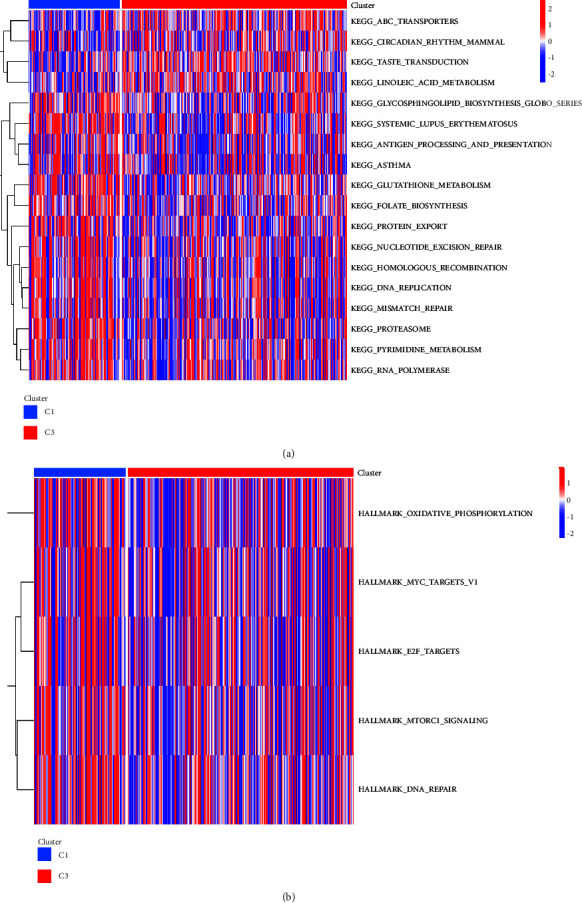
Genomic variation analysis of pathways in C1 and C3 cluster. (a) Heatmap showing the differentially expressed pathways between C1 and C3 cluster based on KEGG pathway enrichment analysis. (b) Heatmap showing the differentially expressed pathways between C1 and C3 cluster based on HALLMARK pathway enrichment analysis.

Cuproptosis is closely associated with prognosis in solid malignancies [5, 20, 21]. Based on the above hypothesis, we stratified samples with qualitatively different CRC based on the expression of 19CRGs via consensus clustering analysis. The results showed that we identified three different clusters of modified patterns, including 100 cases in cluster 1 (with high CRGs and namely C1), 197 cases in cluster 2 (with medium CRGs and namely C2), and 246 cases in cluster 3 (with low CRGs and namely C3) (Figure 2(a) and Supplementary Figure 1). The further survival analysis showed that C1 had a worse survival advantage than C2 and C3 (Figure 2(b)). In addition, MCP counter algorithm was used to calculate the infiltration of 9 immune cells in the three molecular subtypes of CRC, and the differences were analyzed [22]. The results showed that the abundance of B cells, NK cells, and fibroblasts in C2 was higher than that in C3, the abundance of monocytes and CD8^+^T cells in C3 was lower than that in C1 and C2, the abundance of bone marrow dendritic cells and cytotoxic lymphocytes in C2 was higher than that in C1 and C3, and there was no difference in neutrophils and endothelial cells in C1, C2, and C3 group (Figures 2(c)–2(k)). Next, we compared the enrichment differences of KEGG and HALLMARK signaling pathways in C1 and C3 groups by GSVA analysis, and the heat map showed that all pathways with statistical significance were enriched in the C1 group (Figures 3(a) and 3(b)). These results strongly suggest that CRGs may participate in the immune function of CCR via multiple signaling pathways.

### 3.3. Constructing and Evaluating a Risk Score Model Based on CRLs in CRC

**Figure 4:**
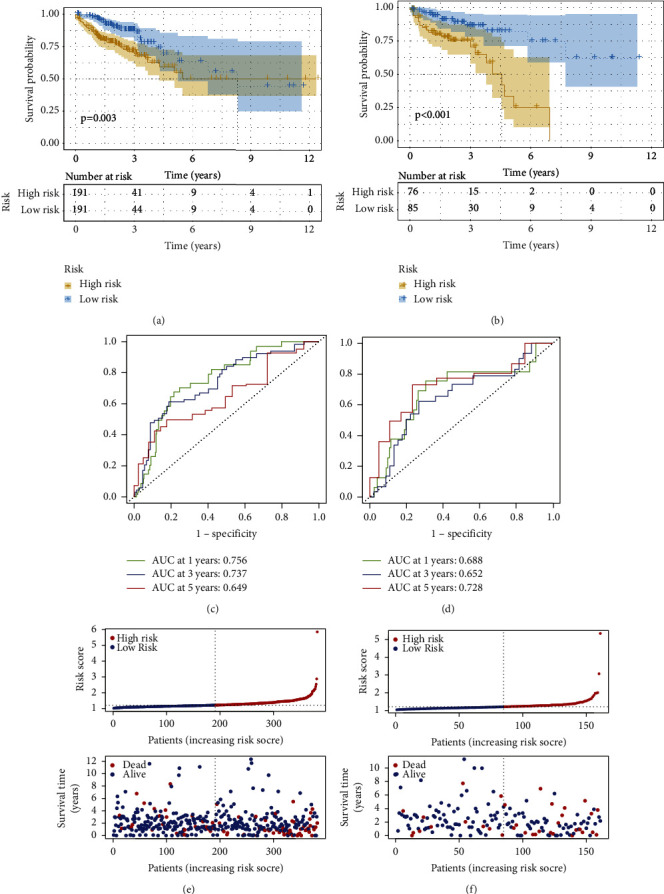
Establishment and validation of a six-gene risk score model (a), (b) Kaplan-Meier curves of overall survival for the high and low-risk groups in the training and validation datasets. (c), (d) Time-dependent receiver operating characteristic curves for the risk score in the training and validation datasets. (e), (f) Distribution of risk scores and survival status for the training and validation datasets.

A variety of studies showed that prognostic models based on lncRNAs have guiding significance for patient prognosis [23]. Therefore, the purpose of this study is to establish a CRLs-based model to facilitate the prognostic prediction of CRC. We previously obtained 33 CRLs with prognostic values, which were further screened by LASSO regression. A total of 6 lncRNAs were obtained, and their risk coefficients were calculated. Specifically, the risk score model for predicting CRC prognosis based on 6 CRLs is shown as follows: risk score = expression value of AC009315.1 *∗* 0.15835365544805 + expression value of PLS3-AS1 *∗* 0.100587632623172 + expression value of ZEB1-AS1 *∗* 0.0274302502732273 + expression value of AC007608.3 *∗* 0.0549982300165668 + expression value of AC010789.2 *∗* 0.166645095217608 + expression value of AC010207.1 *∗* 0.403357464363707. Next, we randomly divided the CRC cohort patients into two cohorts, a training cohort (*n* = 382) and a validation cohort (*n* = 161), in a 7 : 3 ratio. In the training and validation cohort, the patients were divided into low-risk and high-risk groups based on the median risk score. Surprisingly, we found that the low-risk group had a higher survival rate than the high-risk group, both in the training and validation cohort (Figures 4(a) and 4(b)). The AUC values of the 1-year, 3-year and 5-year ROC curves of the training cohort were 0.756, 0.737, and 0.649, respectively, while the AUC values of the 1-year, 3-year and 5-year ROC curves of the validation cohort were 0.688, 0.652, and 0.728, respectively, (Figures 4(c) and 4(d)). In addition, in the training and validation cohort, the signature divided the integrated cohort into low-risk and high-risk groups based on the median risk score (Figures 4(e) and 4(f)). The above results indicated that the risk score model based on CRLs has a good predictive efficiency for the prognosis of CRC patients.

### 3.4. The Correlation Analysis between the Clinical Features and CRLs Risk Score Model for CRC Patients

**Figure 5:**
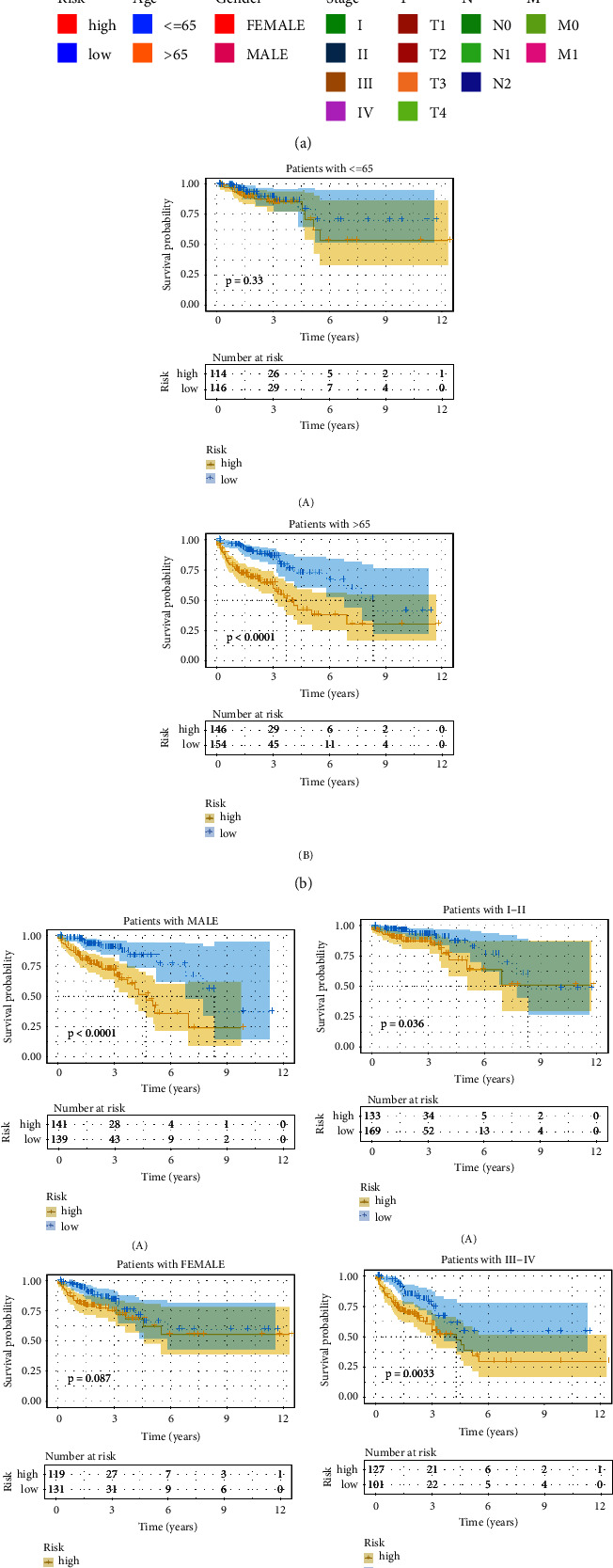
The correlation analysis between the clinical features and CRLs risk score model for CRC patients. (a) Clinicopathological information of CRC patients is arranged by increasing risk score. (b) KM survival curve of high and low risk groups in patients with age ≤65 (A) and age >65 (B). (c) KM survival curves of high and low risk groups in male (A) and female (B) patients. (d) KM survival curves of high and low risk groups for stage I-II (A) and III-IV (B) patients.

We previously established a risk score model based on CRLs and found that it could accurately predict the survival prognosis of CRC patients. To further explore the value of this model, we analyzed the correlation of this model with the clinical features (age, gender and stage) of CRC patients (Figure 5(a)). The results showed that the low-risk group had better survival prognosis in patients aged >65 years, male, stage I-II, stage III-IV, *T*3-4, *N*1-2, and *M*0. There was no difference in survival prognosis between high and low-risk groups in patients aged ≤65 years, female, *T*1-2, *N*0, and *M*1 (Figures 5(b)–5(d)).

### 3.5. Enrichment and Drug Sensitivity Analysis of CRLs Risk Score Model

**Figure 6:**
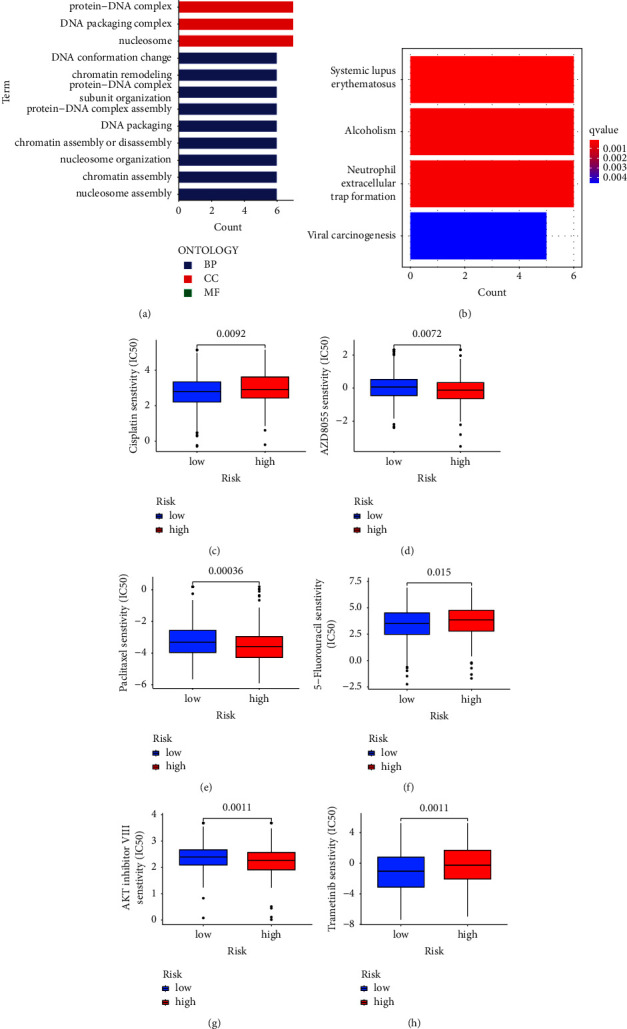
GO and KEGG enrichment and drug sensitivity analysis between high-risk and low-risk groups. (a) GO analysis for the high-risk group and low-risk group. (b) KEGG analysis for the high-risk group and low-risk group. (c) Differences in chemosensitivity to cisplatin in high and low risk groups. (d) Differences in chemosensitivity to AZD8055 in high and low risk groups. (e) Differences in chemosensitivity to paclitaxel in high and low risk groups. (f) Differences in chemosensitivity to 5-Fluorouracil in high and low risk groups. (g) Differences in sensitivity to AKT inhibitor III treatment between high and low-risk groups. (h) Differences in sensitivity to trametinib treatment between high- and low-risk groups. Sensitivity to chemotherapeutic drugs is expressed by the half inhibitory centration (IC50) of chemotherapeutic drugs.

In order to clarify the specific mechanism of the CRLs risk score in CCR, we further analyzed the potential functional pathway of the high-risk and low-risk groups. The results showed that the differentially expressed genes in the high-risk group and the low-risk group were mainly enriched in multiple signaling pathways, such as DNA packaging, chromatin assembly and neutrophil extracellular trap formation (Figures 6(a) and 6(b)). In addition, we further analyzed the association between the CRLs risk score and the efficacy of chemotherapy in the treatment of CRC. It showed that the high-risk group was associated with lower half inhibitory centration (IC50) of chemotherapeutic drugs, such as AZ8055, Paclitaxel, and AKT inhibitor VII, while the IC50 of Cisplatin, 5-Fu, and Trametinib was higher (Figures 6(c)–6(h)). The results showed that the CRLs risk score model could be used as a predictor of chemical sensitivity in the future.

### 3.6. The Relationship between TME and CRLs Risk Score in CRC

**Figure 7:**
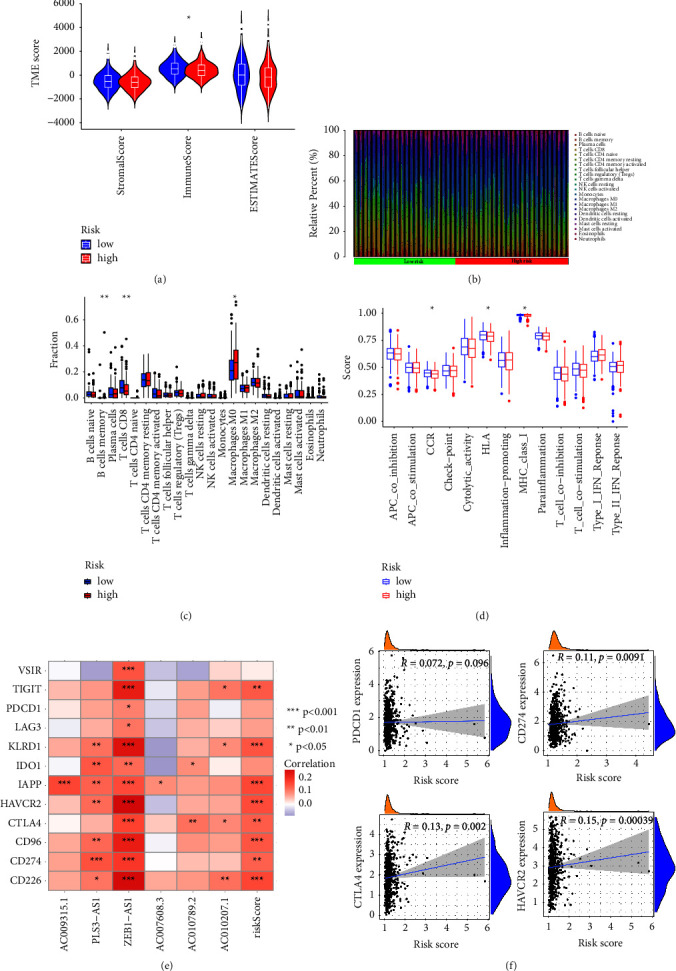
The relationship between the immune landscape and CRLS in CRC. (a) Differences in ESTIMATE score, immune score, and stromal score between high- and low-risk groups. (b), (c) Landscape of immune cell infiltration in high-risk and low-risk groups. (d) Differences in immune cell scores between high-risk and low-risk groups in the CRLS risk score model. (e) Correlation analysis of immune checkpoints and 6 lncRNA expression profiles. (f) Relationship of risk score with PDCD1, CD274, CTLA4, and HAVCR2 expression.

The immune microenvironment of tumors is closely related to tumor progression. Tumor cells interact with immune cells, thereby inhibiting the function of immune cells and finally leading to tumor immune escape [24, 25]. Therefore, we continued to investigate whether the CCR immune microenvironment was associated with CRLs risk scores. We assessed the immune microenvironment of CRC by the ESTIMATE algorithm and observed the differences in the stromal score and immune score between the high-risk group and the low-risk group. As shown in Figure 7(a), lower immune scores were exhibited in the high-risk group. The distribution of 22 immune cells in CRC patients is shown in Figure 7(b). Next, we further calculated the infiltration abundance of immune cells by the CIBORESORT algorithm. The results showed that the infiltrating abundance of CD8^+^ T cells in the low-risk group was higher than that in the high-risk group (Figure 7(c)). Moreover, the boxplot of immune function analysis showed that the scores of chemokine receptors, HLA and MHC in the high-risk group were significantly lower than those in the low-risk group (Figure 7(d)). Immune checkpoints are important predictors for assessing immunotherapy response [26]. Therefore, we evaluated the association of 12 immune checkpoints with CRLs. As shown in Figure 7(e), all lncRNAs in the CRLs risk model were positively associated with most immune checkpoints. Finally, we analyzed the relationship of four common immune checkpoints with risk scores, and the results showed that CD274 (PD-L1), CTLA4, and HAVCR2 (TIM3) were positively associated with risk scores (Figure 7(f)). The above data strongly suggested that CRGs play an important role in the regulation of the CCR immune microenvironment.

### 3.7. Correlataion between Immunotherapy Responsiveness and CRLs Risk Score

**Figure 8:**
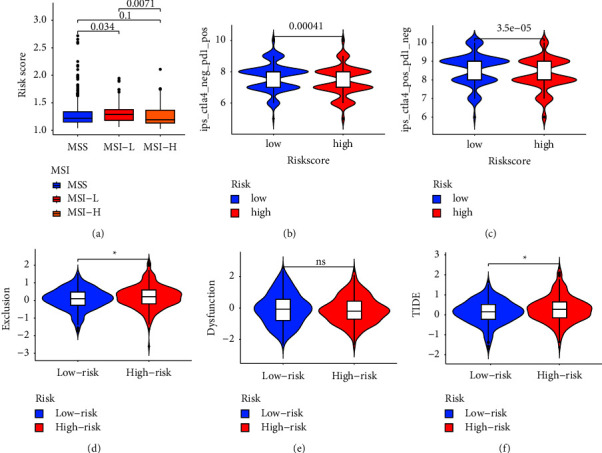
Correlation between immunotherapy responsiveness and CRLs risk score model. (a) Association of MSI status and risk score. (b) IPS scores between high and low CRLs score groups when CTLA‐4 negative and PD1 positive. (c) IPS scores between high and low CRLs score groups when CTLA‐4 positive and PD1 negative. (d‐f) The differences in immune exclusion, immune dysfunction, and TIDE scores between high-risk and low-risk groups.

MSI is an important indicator for evaluating the efficacy of immunotherapy in CRC [27]. Therefore, we explored the association of MSS, MSI-L and MSI-H with CRLS scores. The result showed that MSI-H patients had lower risk scores than MSI-L patients (Figure 8(a)). In recent years, IPS and TIDE have been widely used to evaluate the efficacy of immunotherapy [28, 29]. Our analysis revealed that IPS scores were significantly higher in the low CRLs score group (Figures 8(b) and 8(c)). Consistently, the low-risk group had a lower immune exclusion, immune dysfunction and TIDE scores than the high-risk group (Figures 8(d)–8(f)). These findings indirectly suggest that CRLs may play a key role in mediating immune responses in CRC.

## 4. Discussion

In recent years, the gradual increase in the incidence of CRC has attracted many researchers to lucubrate its occurrence, development and treatment. The resistance of tumors to antitumor therapy has made people gradually realize the importance of programmed cell death, such as autophagy, pyroptosis and ferroptosis [30–32]. Cuproptosis is a newly discovered type of cell death that can be induced by a variety of drugs [33]. Therefore, a full understanding of the specific mechanisms of cuproptosis is critical to guide the treatment of CCR.

In this study, firstly, we found that CRGs were closely associated with CCR immune cell infiltration. Next, we identified 6 CRLs significantly associated with prognostic and then developed a prognostic model. In addition, we validate the accuracy of the CRLs model and initially explore its underlying mechanisms. Finally, we evaluated the relationship between risk scores and immune cell infiltration, drug sensitivity, and immunotherapy efficacy.

In the past decade, more and more studies attempted to establish lncRNA-based prognostic models in order to provide guidance for the prognosis of various malignant tumors. Tang et al. analyzed the expression of ferroptosis-related lncRNAs in head and neck squamous cell carcinoma in a public database, constructed a prognostic model, and further confirmed that it has a good predictive effect. The AUC area for 1 year, 3 years, and 5 years is 0.78, 0.83, and 0.71, respectively [34]. Song et al. analyzed the expression of pyroptosis-related lncRNAs in lung cancer tissues and constructed a prognostic model with good predictive ability. The AUC area for 1 year, 3 years and 5 years is 0.757, 0.728, and 0.685, respectively [35]. In this study, the areas under the AUC curve of our prognostic model at 1 year, 3 years, and 5 years were 0.756, 0.737, and 0.649, respectively. Compared with previous studies, this model shows no weak predictive ability and has good clinical application value.

The immune microenvironment of tumors is regulated by a variety of cells, including tumor cells themselves, immune cells, and fibroblasts [36]. Among them, immune cells play a major role in regulating the tumor immune microenvironment [37]. In recent years, efforts have been made to explore new approaches to treat CCR. The advent of immunotherapy has brought new hope to this idea. A variety of evidence indicates that infiltrating lymphocytes play an important role in the prognosis of various solid tumors and have potential predictive value [38]. In this study, we found that different groups of CRGs have differences in the distribution of immune cells, which indirectly suggests the existence of a relationship between CRGs and the immune microenvironment. More interestingly, we constructed a prognostic model based on CRLs and also showed an association between the risk score and the proportion of immune cells in the tumor microenvironment. This result further supported the relationship between cuproptosis and the immune microenvironment of CCR. However, its specific mechanism needs to be further studied in the future.

Cuproptosis is a novel mode of cell death for which research is currently rather limited. In this study, we found that there were significant differences in the infiltration of various immune cells under different patterns of CRLs. More interestingly, the risk scores of the prognostic models constructed based on CRLs were also significantly different from the immune microenvironment of CRC and its multiple immune checkpoints. These data strongly suggested that there is a strong interrelationship prior to cuproptosis and immunity. Given the existence of an extremely complex network of molecular interactions within cells. In addition, there are some unsatisfactory aspects of this study. Firstly, all data in this study were obtained from public databases, lacking further support from clinical data. Secondly, the mechanism by which the CRLs model regulates the immune microenvironment has not been thoroughly investigated. These issues deserve further research in the future.

In conclusion, in this study, we revealed multiple roles of CRGs and CRLs in CCR. Firstly, CRGs were closely related to CCR immune cell infiltration. Secondly, the risk scoring model based on CRLs has a good predictive ability for the overall survival of CCR. In addition, the risk score of CRLs might have potential guiding value for the application of various antitumor drugs. Moreover, the risk score of CRLs was closely related to the immune cell infiltration of CCR. Finally, the CRLs risk model might have potential instructive value for immunotherapy.

## Data Availability

The data and result in this study are available from the corresponding author for reasonable request.

## Abbreviations

CCR: Colorectal cancer
CRGs: Cuproptosis-related genes
CRLs: Cuproptosis-related lncRNA
KEGG: Kyoto encyclopedia of genes and genomes
TCGA: The cancer genome atlas.

## Supplementary Materials

Supplementary Materials Supplementary Figure 1. Unsupervised clustering of 19 cuproptosis genes in the TCGA cohort. Supplementary Figure 2. Identification of the prognostic genes based on CRLs by LASSO regression. Supplementary Figure 3. The correlation between the clinical features and risk score of CRC patients.
