# Supplementary material for: Construction of a Prognostic Model for Predicting Colorectal Cancer Prognosis and Response to Immunotherapy Based on Cuproptosis-Associated lncRNAs
Source: J Oncol. 2023 Mar 15;2023:2733232. doi: 10.1155/2023/2733232 (PMC10033210; doi:10.1155/2023/2733232)
Supplement: Supplementary Materials — Supplementary Figure 1. Unsupervised clustering of 19 cuproptosis genes in the TCGA cohort. Supplementary Figure 2. Identification of the prognostic genes based on CRLs by LASSO regression. Supplementary Figure 3. The correlation between the clinical features and risk score of CRC patients. [file 2733232.f1.zip › Supplementary Figure 3.pdf]

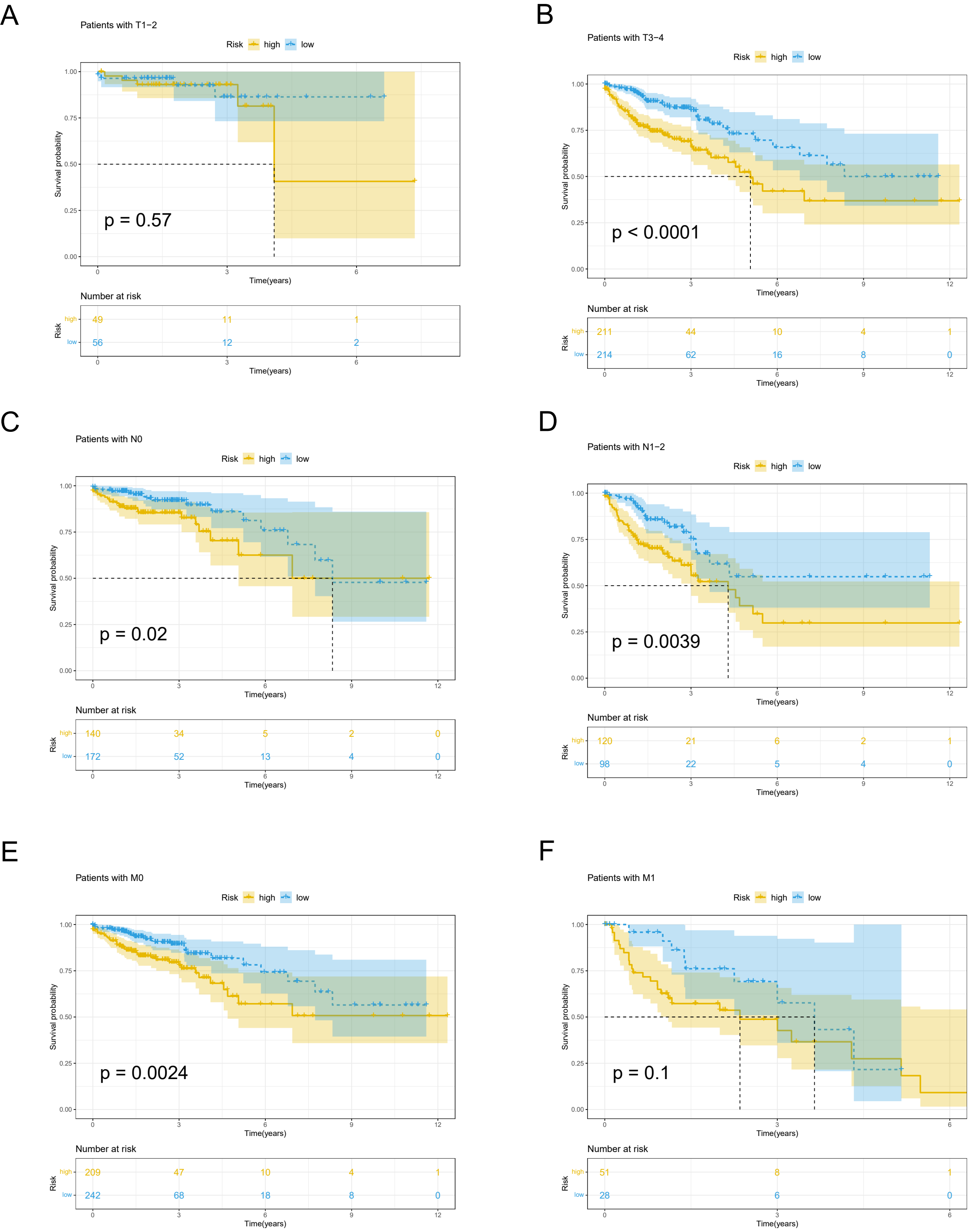

A
Patients with T1−2
Risk high low
Survival probability
1.00
0.75
0.50
0.25
0.00
p = 0.57
0
3
6
Time(years)
Number at risk
Risk
high 49 11 1
low 56 12 2
B
Patients with T3−4
Risk high low
p < 0.0001
0
3
6
9
12
Number at risk
high 211 44 10 4 1
low 214 62 16 8 0
C
Patients with N0
Risk high low
p = 0.02
Number at risk
high 140 34 5 2 0
low 172 52 13 4 0
D
Patients with N1−2
Risk high low
p = 0.0039
Number at risk
high 120 21 6 2 1
low 98 22 5 4 0
E
Patients with M0
Risk high low
p = 0.0024
Number at risk
high 209 47 10 4 1
low 242 68 18 8 0
F
Patients with M1
Risk high low
p = 0.1
Number at risk
high 51 8 1
low 28 6 0
