# Supplementary figures and images for: Construction of a Prognostic Model for Predicting Colorectal Cancer Prognosis and Response to Immunotherapy Based on Cuproptosis-Associated lncRNAs
Source: J Oncol. 2023 Mar 15;2023:2733232. doi: 10.1155/2023/2733232 (PMC10033210; doi:10.1155/2023/2733232)

A

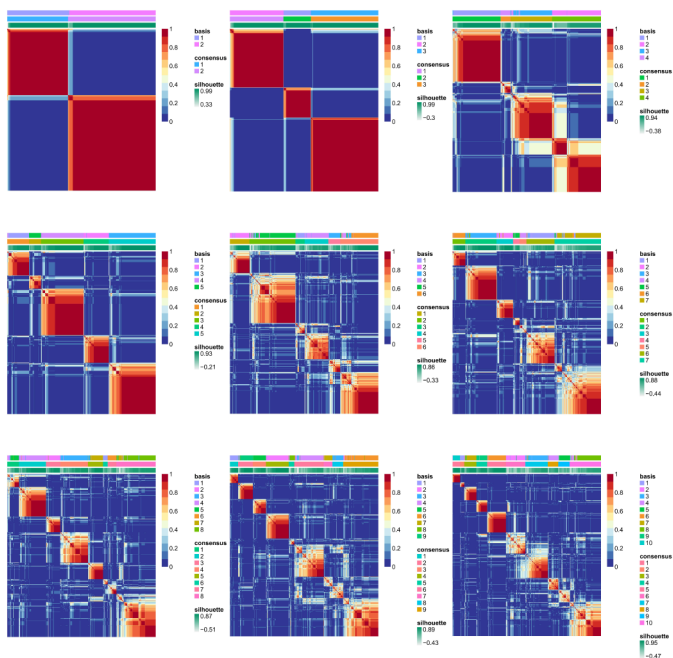

B

NMF rank survey

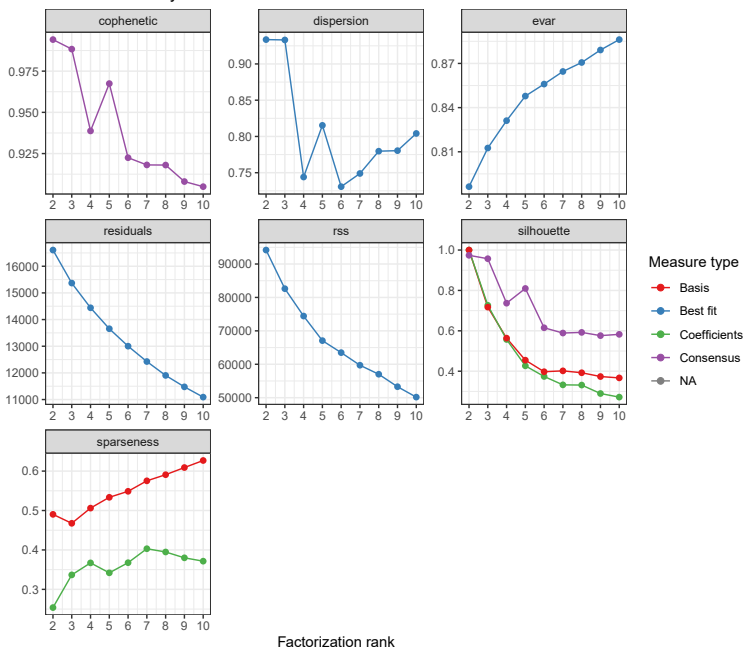

Supplement: Supplementary Materials — Supplementary Figure 1. Unsupervised clustering of 19 cuproptosis genes in the TCGA cohort. Supplementary Figure 2. Identification of the prognostic genes based on CRLs by LASSO regression. Supplementary Figure 3. The correlation between the clinical features and risk score of CRC patients. [file 2733232.f1.zip › Supplementary Figure 1.pdf]

A

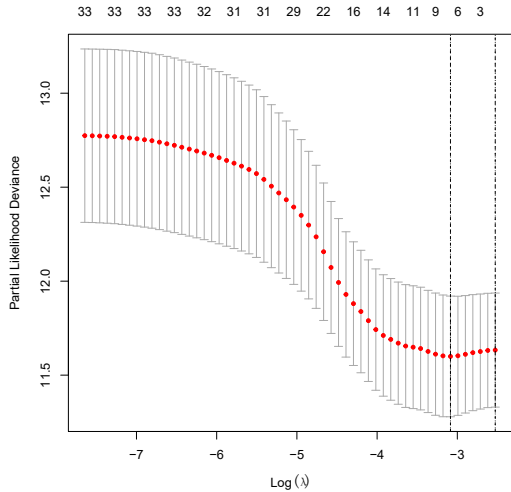

B

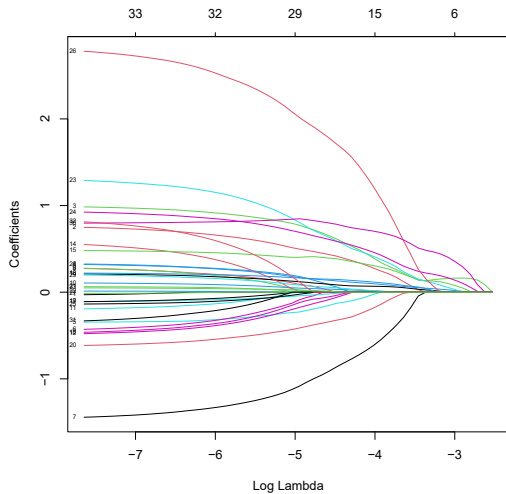

Supplement: Supplementary Materials — Supplementary Figure 1. Unsupervised clustering of 19 cuproptosis genes in the TCGA cohort. Supplementary Figure 2. Identification of the prognostic genes based on CRLs by LASSO regression. Supplementary Figure 3. The correlation between the clinical features and risk score of CRC patients. [file 2733232.f1.zip › Supplementary Figure 2.pdf]
