# Supplementary material for: Construction of a Prognostic Model for Predicting Colorectal Cancer Prognosis and Response to Immunotherapy Based on Cuproptosis-Associated lncRNAs
Source: J Oncol. 2023 Mar 15;2023:2733232. doi: 10.1155/2023/2733232 (PMC10033210; doi:10.1155/2023/2733232)
Supplement: Supplementary Materials — Supplementary Figure 1. Unsupervised clustering of 19 cuproptosis genes in the TCGA cohort. Supplementary Figure 2. Identification of the prognostic genes based on CRLs by LASSO regression. Supplementary Figure 3. The correlation between the clinical features and risk score of CRC patients. [file 2733232.f1.zip › Supplementary Figures.doc]

**Supplementary Figures**

Supplementary Figure 1 Unsupervised clustering of 19 cuproptosis genes in the TCGA cohort. (A) Heat map of NMF clustering of angiogenic genes with the number of clusters from 2 to 9. (B) Covariance, dispersion, residuals and silhouette coefficients versus the number of clusters.

Supplementary Figure 2 Identification of the prognostic genes based on CRLs by LASSO regression

(A)	The prognostic genes screened by lasso regression analysis. When the number of variables was 6，the value of partial likelihood deviation is the minimum. (B) Cross validation results. The regression coefficient map of CRLs in this LASSO model.

Supplementary Figure 3 The correlation between the clinical features and risk score of CRC patients

(A-F) KM survival curves of high and low risk groups for stage T1-T2, T3-T4, N0, N1-2, M0 and M1patients.
